# Droplet Digital PCR Quantification of Selected Intracellular and Extracellular microRNAs Reveals Changes in Their Expression Pattern during Porcine In Vitro Adipogenesis

**DOI:** 10.3390/genes14030683

**Published:** 2023-03-09

**Authors:** Adrianna Bilinska, Marcin Pszczola, Monika Stachowiak, Joanna Stachecka, Franciszek Garbacz, Mehmet Onur Aksoy, Izabela Szczerbal

**Affiliations:** Department of Genetics and Animal Breeding, Poznan University of Life Sciences, 60-637 Poznan, Poland

**Keywords:** adipocytes, ddPCR, ECmiRNAs, mesenchymal stem cells, pig

## Abstract

Extracellular miRNAs have attracted considerable interest because of their role in intercellular communication, as well as because of their potential use as diagnostic and prognostic biomarkers for many diseases. It has been shown that miRNAs secreted by adipose tissue can contribute to the pathophysiology of obesity. Detailed knowledge of the expression of intracellular and extracellular microRNAs in adipocytes is thus urgently required. The system of in vitro differentiation of mesenchymal stem cells (MSCs) into adipocytes offers a good model for such an analysis. The aim of this study was to quantify eight intracellular and extracellular miRNAs (miR-21a, miR-26b, miR-30a, miR-92a, miR-146a, miR-148a, miR-199, and miR-383a) during porcine in vitro adipogenesis using droplet digital PCR (ddPCR), a highly sensitive method. It was found that only some miRNAs associated with the inflammatory process (miR-21a, miR-92a) were highly expressed in differentiated adipocytes and were also secreted by cells. All miRNAs associated with adipocyte differentiation were highly abundant in both the studied cells and in the cell culture medium. Those miRNAs showed a characteristic expression profile with upregulation during differentiation.

## 1. Introduction

MicroRNAs (miRNAs) are a well-known class of small, noncoding RNAs that regulate post-transcriptional gene expression through mRNA destabilization or inhibition of translation [1]. To date, over 2500 miRNAs have been discovered in the human genome, and it is estimated that they regulate over 60% of protein-coding genes [2]. miRNAs thus play an essential role in all biological processes, including cell differentiation and development [3]. Changes in miRNA expression have been reported in altered physiological conditions and various diseases, so these molecules have been treated as promising therapeutic targets. miRNA-based therapies involve correcting altered miRNA expression levels using mimics or inhibitors [4]. Moreover, miRNAs can be used as biomarkers of pathophysiological conditions [5]. In particular, extracellular miRNAs (ECmiRNAs) can serve as good diagnostic markers due to their stability and ease of sample collection. ECmiRNAs have been detected in cell-free conditions, including cell culture media and biological fluids, such as serum, plasma, saliva, tears, urine, breast milk, etc. [6]. 

The role of miRNAs has been extensively studied in the context of the development of obesity. It has been shown that miRNAs are involved in the control of a range of processes, including adipogenesis, insulin resistance, and inflammation in adipose tissue [7].

Dysregulation of many miRNAs has been identified in the adipose tissue of obese individuals [8,9,10]. The presence of adipocyte-related miRNAs in adipocyte-derived microvesicles indicates their involvement in intercellular communication in both paracrine and endocrine manners [10,11,12]. Studies of miRNA in adipocyte tissue have also been conducted on the domestic pig (*Sus scrofa*), an important animal model for human obesity and also a major livestock species [13]. There are a number of reports on the functioning of individual miRNAs during the formation of fat tissue in the pig (summarized by Song et al. [14]). High-throughput miRNA profiling of porcine adipocyte tissue has also allowed the detection of a complex microRNA–mRNA regulatory network related to fat deposition in pigs [15,16,17,18]. A recent study of the identification of miRNAs in porcine adipose-derived and muscle-derived exosomes showed some miRNAs to be involved in skeletal muscle–adipose crosstalk [19]. 

Most of the research on porcine miRNAs has been carried out on adipose tissues, while studies on in vitro models of adipogenesis are scarce [20]. Due to the heterogeneous nature of adipose tissue—which is composed of several cell types, including adipocytes, preadipocytes, stem cells, endothelial cells, and various blood cells [21]—cultured adipocytes represent a good system for studying molecular events that occur during adipogenesis, including the secretion of miRNA by adipocytes [22]. The aim of this study was thus to quantify eight miRNAs (miR-21a, miR-26b, miR-30a, miR-92a, miR-146a, miR-148a, miR-199a, and miR-383) during porcine in vitro differentiation of mesenchymal stem cells (MSCs) into adipocytes. These miRNAs were selected on the basis of their role in differentiation and inflammation processes (Table 1). The expression of intracellular and extracellular microRNAs was evaluated using droplet digital PCR (ddPCR), a highly sensitive method.

## 2. Materials and Methods

### 2.1. Mesenchymal Stem Cell Culture

Mesenchymal stem cells were derived from the adipose tissue (AD-MSCs) of a three-month-old female Polish Large White pig. Tissue sample collection was approved by the Local Ethical Commission for Experiments on Animals at Poznan University of Life Sciences, Poznan, Poland (approval no. 57/2012). Following Stachecka et al. [39], the AD-MSCs were cultured in Advanced DMEM (Gibco, Life Technologies, Grand Island, NY, USA) supplemented with 10% FBS (*v/v*) (Sigma-Aldrich, St. Louis, MO, USA), 5 ng/mL FGF-2 (PromoCell GmbH, Heidelberg Germany), 2 mM L-glutamine (Gibco), 1 mM 2-mercaptoethanol (Sigma-Aldrich), 1 × antibiotic antimycotic solution (Sigma-Aldrich), and 1 × MEM NEAA (Gibco) at 37 °C in 5% CO_2_. To avoid the possible influence of FBS-derived miRNAs on obtained results, the same part of filtered FBS was used during the whole cell culture experiment. The AD-MSCs were propagated by passaging using standard cell culture procedures, and their stemness was confirmed by staining for positive (CD44, CD90, CD105) and negative (CD45) markers (Abcam, Cambridge, UK).

### 2.2. Adipogenic Differentiation

Adipogenesis was induced by culturing early-passage MSCs in an adipogenic differentiation medium composed of Advanced DMEM (Gibco), 10% FBS (Sigma-Aldrich), 1 × antibiotic antimycotic solution (Sigma-Aldrich), 1 × MEM NEAA (Gibco), 5 ng/mL FGF-2 (PromoCell GmbH), 1 × linoleic acid albumin, 1 × ITS, 1 µm dexamethasone (Sigma-Aldrich), 100 µm indomethacin (Sigma-Aldrich), and 50 mM IBMX (Sigma-Aldrich). The cells were cultured for ten days. Adipogenic differentiation was monitored using visual examination of lipid droplet formation under a phase-contrast microscope (Nikon TS100 Eclipse, Melville, NY, USA) and BODIPY staining. Cells were fixed with 4% paraformaldehyde in PBS (*w/v*) for ten minutes at room temperature and washed thrice with PBS. The cells were then incubated with BODIPY 493/503 (Thermo Fisher, Waltham, MA, USA) in PBS (3 µg/mL) and washed thrice in PBS. The nuclei were counterstained with DAPI in Vectashield medium (Vector Laboratories, Newark, CA, USA) and examined under a fluorescence microscope (Nikon E600 Eclipse, Melville, NY, USA). Each measurement was performed in triplicate. 

### 2.3. RNA Extraction from Cells and Culture Medium

Total RNA extraction from cells (approximately 2 × 10^6^ in number) and the cell culture medium (200 µL) was performed on days 0, 2, 4, 6, 8, and 10 of adipogenesis using the miRNeasy Micro Kit (Qiagen, Hilden, Germany), following the manufacturer’s protocol. The RNA samples isolated from cell culture medium were enriched in the fraction of miRNAs, both exosomal and non-exosomal ECmiRNAs. All samples were analyzed in duplicate. The RNA concentrations and quality were assessed using a NanoDrop 2000 spectrophotometer (Thermo Scientific, Wilmington, DE, USA) and Qubit RNA HS Assay Kit (Thermo Fisher Scientific) on a Qubit 2.0 Fluorometer (Thermo Fisher Scientific). 

### 2.4. Real-Time PCR

One microgram of RNA was reversely transcribed using a Transcriptor High Fidelity cDNA Synthesis kit (Roche Diagnostic, Mannheim, Germany). Primer sets for quantitative real-time PCR for selected protein-coding marker and reference genes (Table S1) were designed using the PRIMER 3 software (http://simgene.com/Primer3 (accessed on 12 May 2022)). The relative transcript levels were assessed using a LightCycler 480 SYBR Green I Master kit (Roche Diagnostic) with a LightCycler 480 II (Roche Life Science). All samples were analyzed in triplicate. Standard curves were designed as tenfold dilutions of the PCR products. Relative transcript levels of the studied genes were calculated after normalization with the transcript level of a reference gene, ribosomal protein L27 (*RPL27*), which has shown stability during adipogenic differentiation [40,41].

### 2.5. miRNA-Specific Reverse Transcription

Reverse transcription was performed with 10 ng of total RNA using a TaqMan MicroRNA Reverse Transcription Kit (Applied Biosystems, Foster City, CA, USA). Reverse transcription reactions were conducted with the use of an RT primer specific to each tested miRNA. The following TaqMan MicroRNA Assays (Applied Biosystems) were employed: miR-21a-5p, (Assay ID: 000397), miR-26b-5p (Assay ID: 000406), miR-30a-5p (Assay ID: 000417), miR-92a-3p (Assay ID: 000431), miR-146a (Assay ID: 005896), miR-148a-3p (Assay ID: 000470), miR-199a-3p (Assay ID: 002304), and miR-383-5p (Assay ID: 000573). RNU6b (Assay ID: 001093) was used as the reference for normalizing the ddPCR results [42]. The reverse transcription reactions were performed following the manufacturers’ recommendations.

### 2.6. Droplet Digital PCR (ddPCR)

miRNA quantification was performed using droplet digital PCR (ddPCR). All samples were analyzed in duplicate. Each PCR reaction consisted of 1 µL cDNA, 11 µL of 2 × ddPCR SuperMix for Probes (Bio-Rad, Hercules, CA, USA), 9 µL of H_2_O, and 1 µL of TaqMan primers and probe from the corresponding TaqMan MicroRNA Assay (Applied Biosystems). The reaction mixtures were divided into approximately 20,000 droplets using a QX200 droplet generator (Bio-Rad) followed by PCR performed on a T100 Thermal Cycler (Bio-Rad) using the following conditions (ramp rate of 2 °C/s): initial denaturation at 95 °C for 10 min, 40 cycles at 94 °C for 30s, followed by 60 °C for 1 min and denaturation at 98 °C for 10 min. A QX200 droplet reader (Bio-Rad) was used to detect fluorescence, and the results were analyzed using QuantaSoft software (Bio-Rad). The fraction of positive droplets was quantified using the Poisson distribution.

Since cell culture media may carry miRNAs derived from supplements such as fetal bovine serum (FBS) [43], an experiment on the expression level of the investigated miRNAs in the pure cell culture medium, supplemented with 10% of FBS, was performed. Expression of miR-92a, miR-146a, and miR-26b was not observed, while expression of miR-21a, miR-383, miR-30a, miR-148a, and miR-199a was on very low level (Table S2), which was about 1% of the average expression level of extracellular miRNAs (Table S6). Thus, an additional normalization step was abandoned.

### 2.7. Statistical Analysis

Differences between expression levels were assessed separately for each miRNA and for each medium. To give the analyzed variables a normal distribution, the expression levels were transformed by taking the natural logarithm of the original values. The following model was then used to assess the differences between the expression levels on each day:

log (*Exp*) *_ij_* = µ + *DAY _j_* + *sampleID _i_* + *error _ij_*,

where log (*Exp*) is the natural logarithm of the expression level recorded on the *j*th *DAY* for the *i*th *sampleID*. *DAY* was a categorical variable with six levels (0, 2, 4, 6, 8, 10). The *sampleID* and *error* were random terms. The *sampleID* was treated as random term to account for repeated observations of the sample on following days. The analyses were performed using the R environment [44]. The effects of the model were estimated using the lme4 package [45] and the significance of the differences between days was assessed using the lmerTest [46] and emmeans packages [47], making use of Satterthwaite’s method [48] for approximating degrees of freedom. The *p*-values for comparing expression levels on particular days were adjusted for multiple comparisons using Tukey’s method for comparing six estimates. 

To assess whether there was a relationship between the expression level in the medium and cells, the previously used model was updated to include the log-transformed expression in the medium log (*Exp_medium_*). The following model was thus used:

log (*Exp*) *_ij_* = µ + log (*Exp_medium_*) *_ij_* + *DAY _j_* + *sampleID _i_* + *error _ij_.*

The regression line was obtained by applying the locally weighted scatterplot smoothing method available from the ggplot2 package [49].

## 3. Results

**Figure 1:**
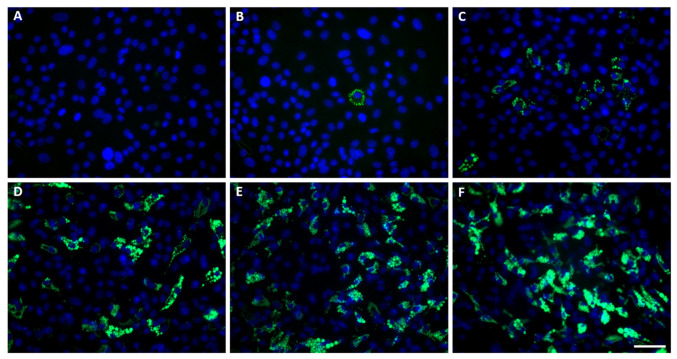
The adipocyte differentiation experiment. Mesenchymal stem cells (MSCs, day 0 of differentiation) were treated with adipogenic hormonal inducers and were cultured for ten days. Cells and media were collected for total RNA isolation on days 0 (**A**), 2 (**B**), 4 (**C**), 6 (**D**), 8 (**E**), and 10 (**F**). Representative images were taken after staining the lipid droplets with BODIPY 493/503 (green); the nuclei were counterstained with DAPI (blue). Scale bar: 50 µm.

Eight miRNAs associated with inflammatory processes (miR-21a, miR-92a, miR-146a, miR-383) and adipocyte differentiation processes (miR-26b, miR-30a, miR-148a, miR-199a) were included in this study (Table 1). The abundances of these miRNAs were determined in cells and in cell culture medium over ten days of adipogenic differentiation (Figure 1).

**Figure 2:**
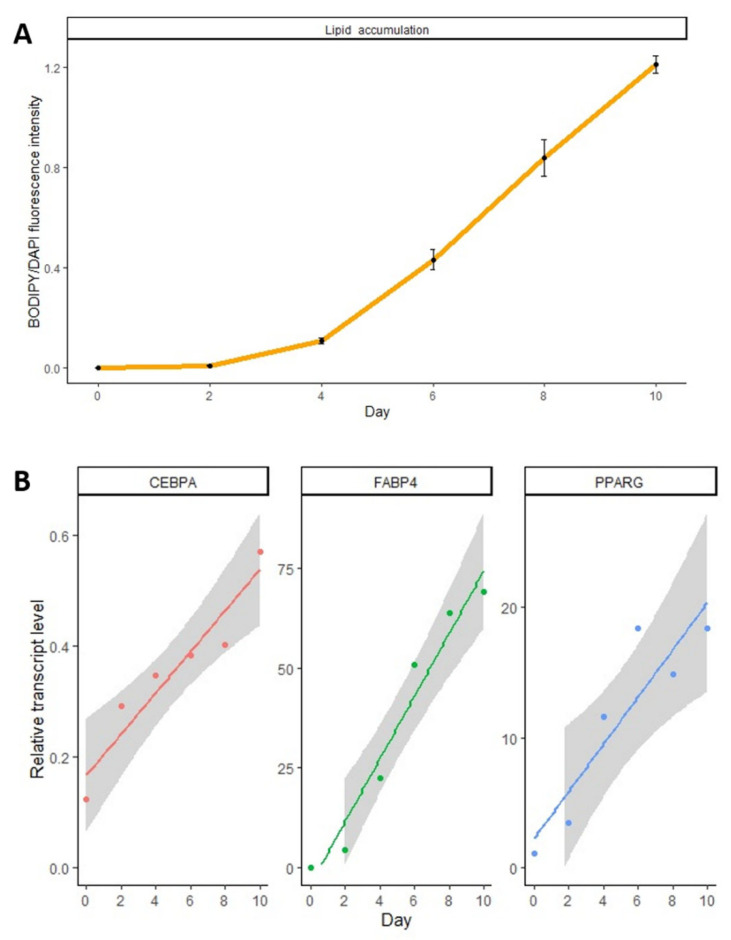
Monitoring of adipocyte differentiation. (**A**) Lipid droplet accumulation was quantified by measuring BODIPY 493/503/DAPI fluorescent intensity. Error bars show SDs. The error bars represent standard deviations. (**B**) Measurements of the relative transcript level of three genes: *CEBPA*, *FABP4*, and *PPARG.* Each dot represents relative expression (mean of 3 repeats). The line is simple linear regression based on the collected data points and the gray area represents the 0.95 confidence interval for the regression line.

The differentiation process was monitored by evaluating the accumulation of lipid droplets using BODIPY staining (Figure 1 and Figure 2A, Table S3). On day 4, individual cells with lipid droplets were seen, while lipid accumulation was highly abundant from day 6. Adipocyte differentiation was also confirmed by the upregulation of expression of three marker genes: *CEBPA*, *FABP4*, and *PPARG* (Figure 2B, Table S3).

**Figure 3:**
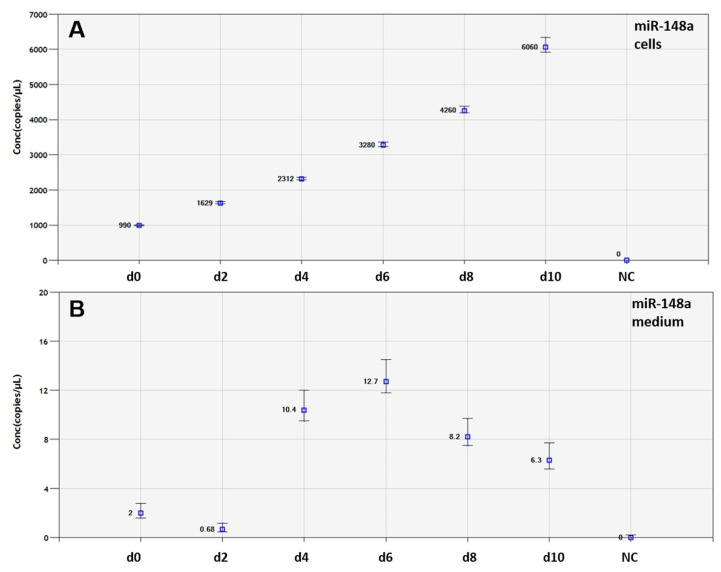
Examples of the detection of miRNAs using the ddPCR method. Absolute quantification (concentration in copies/µL) of miR-148a in cells (**A**) and cell culture medium (**B**) at days 0 (d0), 2 (d2), 4 (d4), 6 (d6), 8 (d8), and 10 (d10). NC: negative control (sample without cDNA template). Error bars indicate the Poisson 95% confidence intervals.

The expression of all the miRNAs was successfully detected with the ddPCR method (Figure 3). 

**Figure 4:**
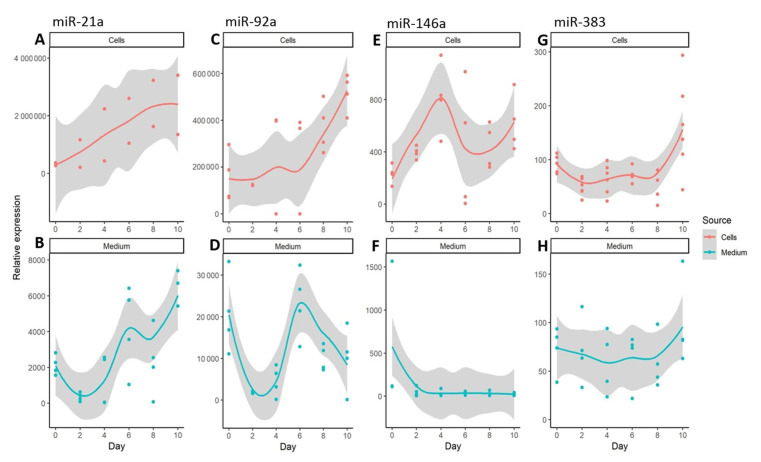
Relative expression levels of extracellular and intracellular miRNAs related to inflammation during the ten days of adipocyte differentiation. The expression of miR-21a (**A**,**B**), miR-92a (**C**,**D**), miR-146a (**E**,**F**), and miR-383 (**G**,**H**) was normalized using RNU6B. Dots represent the actual measurements, the line is a local regression based on the collected data points, and the gray area represents the 0.95 confidence interval for the regression line.

It was found that, of the miRNAs associated with the inflammatory process, miR-21a showed the highest expression in differentiated adipocytes and was also highly secreted by these cells (Figure 4A,B; Tables S4 and S5). Both intracellular and extracellular miR-21a levels were upregulated during adipogenesis. miR-92a was also highly expressed by adipocytes, reaching its highest level on day 10 of differentiation. The abundance of extracellular miR-92a initially decreased on days 2–4, returned to its original level after day 6, and then decreased (Figure 4C,D; Tables S4 and S5). The expression of miR-146a in the studied differentiation system was quite low (Figure 4E,F; Tables S4 and S5). Cellular miR-146a was upregulated during adipogenesis, but was not secreted by the differentiated cells. The lowest expression level was found for miR-383, and this was comparable in the cells and in the cell culture medium (Figure 4G,H; Tables S4 and S5).

**Figure 5:**
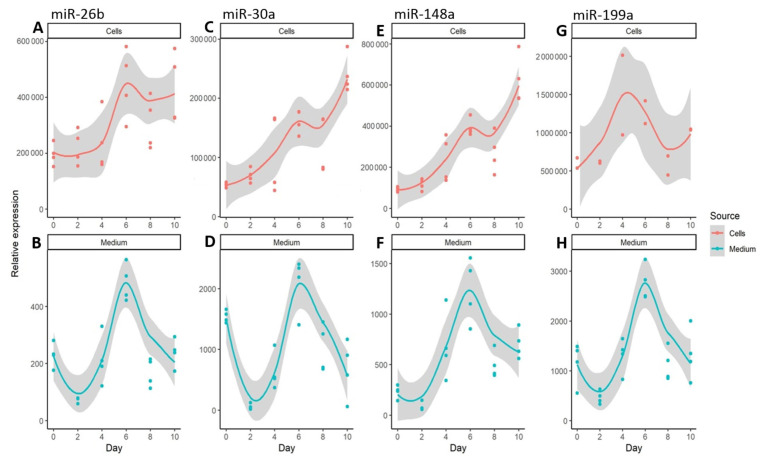
Relative expression levels of extracellular and intracellular miRNAs related to adipogenesis during the ten days of adipocyte differentiation. The expression of miR-26b (**A**,**B**), miR-30a (**C**,**D**), miR-199a (**E**,**F**), miR-148a (**G**,**H**) was normalized using RNU6B. Dots represent the actual measurements, the line is a local regression based on the collected data points, and the gray area represents the 0.95 confidence interval for the regression line.

In terms of miRNAs associated with adipogenesis, all the molecules we examined here were highly expressed during differentiation (Figure 5; Tables S4 and S5). The highest expression in cells was found for miR-26b, next to miR-199a, miR-148a, and miR-30a. Of these, miR-26b, miR-148a, and miR-30a had the highest expression levels at the end of differentiation (day 10), while for miR-199a this occurred on day 4 of adipogenesis. All extracellular miRNAs had similar expression profiles, reaching the highest level on day 6 of differentiation. miR-199a, miR-30a, and miR-148a were secreted at comparable levels, while the amount of miR-26b was lower.

Comparing the expression levels of intracellular and extracellular miRNAs showed higher expression in cells than in the medium for all the studied miRNAs except miR-383 (Table S6). No relationship was found between the expression level of intracellular and extracellular miRNAs (Table S7).

## 4. Discussion

To better understand the role of miRNA in adipocyte formation, we examined the expression of the selected intracellular and extracellular miRNAs during adipogenesis, using the domestic pig as a model organism. We employed ddPCR, as a robust method for absolute quantification of miRNAs [50]. This method has proven to be especially useful for quantifying extracellular miRNAs [51,52]. Here, we showed the usefulness of ddPCR for detecting less abundant ECmiRNAs in adipogenic spent medium. 

We found that, of the studied miRNAs, miR-21a showed the highest expression in differentiated cells, and its expression was very high in the cell culture medium. It has been reported that miR-21 is frequently upregulated in many chronic diseases, including obesity [25]. It plays a pivotal role in the functioning of adipose tissue through its regulation of many biological processes, such as thermogenesis, browning of adipose tissue, angiogenesis, apoptosis, and adipogenesis [53]. A previous study of MSCs isolated from human adipose tissue showed that miR-21 expression increased in the early stages of adipogenic differentiation and gradually decreased after day 3 [24]; in our differentiation system, miR-21a was upregulated throughout the entire differentiation period. Studies of miR-21 mimics and inhibitors as therapeutic agents in obesity treatment have also provided varying results [53,54]. miR-21 has also been depicted as secreted by macrophages of adipose tissue [10], while in this study we confirmed its secretion by adipocytes. Further studies, including of both the intracellular and extracellular form of miR-21a, are thus needed. 

To date, little has been learned about the role of miR-92a in adipogenesis. There are reports of its involvement in brown adipocyte differentiation [55]. Exosomal miR-92a abundance has also been observed in human serum after cold-induced brown adipose tissue activity [56]. In 3T3L1 cells, the miR-17–92 cluster accelerated adipocyte differentiation by negatively regulating the tumor suppressor Rb2/p130 during the early stages of adipogenesis [57]. Here, we provide evidence that miR-92a alone is upregulated during porcine adipogenesis and is secreted by adipocytes.

Recently, miR-146a has been recognized as a potential regulator of porcine intramuscular preadipocytes [58]. The authors reporting this observed that miR-146a-5p mimics inhibited preadipocyte proliferation and differentiation, while the miR-146a-5p inhibitors promoted cell proliferation and adipogenic differentiation. Both that study and our present one found a similar expression pattern for these miRNAs, with the expression peaking in the early stages of adipogenesis (on days 2 or 4 of differentiation, respectively). Interestingly, miR-146a was one of the studied miRNAs that was not secreted by differentiated cells (as was miR-383). 

Of the studied miRNAs, miR-26b, miR-30a, miR-148a, and miR-199a have been reported as involved in adipocyte formation through their promotion or acceleration of adipogenesis, which they achieve by regulating numerous target genes [27,28,34,59]. Their expression was found to gradually increase after the induction of adipocyte differentiation, as in our study. Only miR-199a reached its highest expression at day 4 of adipogenesis, and its expression then decreased, as confirmed by its function in the proliferation and differentiation of porcine preadipocytes [60]. This miRNA was highly expressed in cells and also secreted more. It seems that this molecule has a comprehensive set of functions and plays a role in a range of different processes, such as angiogenesis, aging, apoptosis, proliferation, and myogenic differentiation [61,62]. 

All the extracellular miR-26b, miR-30a, miR-148a, miR-199a, and miR-92a showed the same expression profiles, with their expression peaking on day 6 of differentiation. It was previously reported that exosomal miRNAs are secreted from hypertrophic adipocytes and transferred to small adipocytes to promote lipogenesis and hypertrophy of emerging adipocytes [63,64]. This may be one reason why high expression of these extracellular miRNAs is observed in the intermediate days of differentiation, when new adipocytes arise at an intense rate. As we found no strong correlation between the expression of intracellular and extracellular miRNAs, it can be anticipated that the secretion of miRNA is an independent process regulated by other mechanisms, such as the formation of extracellular vesicles such as exosomes or transportation via protein–miRNA complexes [65,66]. 

Our study revealed relatively high intercellular variation of miRNA expression (Tables S4–S6, which may be related to the heterogeneity of cell populations in terms of differentiation timing. It has been shown in previous studies that a high standard deviation is found for low-expressed miRNA [67]. Application of new methods, such as single-cell microRNA–mRNA co-sequencing, revealed that microRNA expression variability might be responsible alone for non-genetic cell-to-cell heterogeneity [68]. The authors found that miRNAs with low expression levels showed inherently large standard deviations, while the variation of high-abundance miRNAs gradually decreased as the expression level increased. 

It has been shown that the miRNA expression profile can serve as a signature of cell identity, through the expression of a unique miRNA. However, this would seem to be difficult to apply to adipose tissue, as it expresses a wide range of miRNAs [65,69]. Our study of cultured adipocytes allowed us to obtain more detailed knowledge of the relationship between the intracellular and extracellular microRNAs that are expressed during the formation of adipocyte cells. Taking into account the fact that miRNAs from adipose tissue participate in intercellular and interorgan communications, and that their aberrant expression may lead to pathological conditions, further comprehensive studies of extracellular and intracellular miRNAs are needed. 

## 5. Conclusions

We showed that miRNAs associated with adipogenesis and inflammation processes are expressed by differentiated adipocytes. Both intracellular and extracellular miRNAs have characteristic expression profiles during porcine adipogenesis. We found that there is no relationship between the expression level of intracellular miRNAs and the levels of extracellular miRNAs. ddPCR proved a useful method of quantifying miRNAs during in vitro adipogenesis, especially for less abundant extracellular miRNAs.

## Figures and Tables

**Table 1:** Characteristics of the analyzed miRNAs.

microRNA	Function	Reference
miR-21a	modulates inflammation, regulates adipogenic differentiation, and is upregulated in obesity	[23,24,25]
miR-26b	mediates the multiple differentiation of MSCs and promotes adipocyte differentiation	[26,27]
miR-30a	accelerates adipogenesis and promotes fatty acid and glucose metabolism in adipocytes	[28,29]
miR-92a	controls inflammatory response and inhibits adipose browning	[30,31]
miR-146a	plays a role in inflammatory process in various disorders and is upregulated in obesity	[32,33]
miR-148a	regulates MSC differentiation into adipocytes, a biomarker of obesity	[34,35]
miR-199a	regulates adipocyte differentiation and fatty acid composition during adipogenesis	[36,37]
miR-383	its expression correlates with various inflammatory diseases	[38]

## Data Availability

The data supporting the findings of the study are available from the corresponding author (I.S.), upon reasonable request.

## Supplementary Materials

The following supporting information can be downloaded from https://www.mdpi.com/article/10.3390/genes14030683/s1. Table S1: PCR primers used to study the expression level of adipocyte differentiation marker genes. Table S2: Expression levels of miRNAs in cell culture medium supplemented with 10% of fetal bovine serum (FBS). SE—standard error; Table S3: Lipid droplet content and relative gene expression of *CEBPA, FABP4*, and *PPARG* genes across the consecutive days of experiment (supplementary to Figure 2). The regression slope and *p*-values were obtained using the locally weighted scatterplot smoothing method of the ggplot2 package. Table S4: Expression levels of intracellular miRNAs over the course of differentiation. The comparisons were made between days of the experiment for one miRNA at a time, and so the table should be read column by column. The same letters indicate an absence of significant difference (at the 0.05 level) between the expression levels; Table S5: Expression levels of extracellular miRNAs over the course of differentiation. The comparisons were made between days of the experiment for one miRNA at the time, so the table should be read column by column. The same letters indicate an absence of significant difference (at the 0.05 level) between the expression levels; Table S6: Expression levels of intracellular and extracellular miRNAs. The comparisons were made between miRNAs, so the table should be read row by row. The same letters indicate an absence of significant difference (at the 0.05 level) between the expression levels; Table S7: Relationship between the expression in the medium and cells based on regression slopes for the log-transformed expression in the medium on the log-transformed expression in cells for different miRNA.
