# Supplementary material for: Droplet Digital PCR Quantification of Selected Intracellular and Extracellular microRNAs Reveals Changes in Their Expression Pattern during Porcine In Vitro Adipogenesis
Source: Genes (Basel). 2023 Mar 9;14(3):683. doi: 10.3390/genes14030683 (PMC10047974; doi:10.3390/genes14030683)
Supplement: Supplementary file 1 [file genes-14-00683-s001.zip › genes-2133091-supplementary.pdf]

Supplementary Material

Droplet digital PCR quantification of selected intracellular and extracellular microRNAs reveals changes in their expression pattern during porcine in vitro adipogenesis

Adrianna Bilinska, Marcin Pszczola, Monika Stachowiak, Joanna Stachecka, Franciszek Garbacz, Mehmet Onur Aksoy, Izabela Szczerbal*

Table S1. PCR primers used to study the expression level of adipocyte differentiation marker genes.

Gene	Primer sequence	Product size (bp)	Annealing temperature (ºC)	Genbank accesion number
RPL27	F: 5` GCAAAGCGGTCATCGTAAA<br>R: 5` CTTGTGGGCATGAGGTGAT	190	60	NM_001097479.1
PPARG	F: 5` GCATCAGCTCTGTGGACCTG<br>R: 5` GATCAGCTCTCGGGAATGGG	132	60	XM_005669783
CEBPA	F: 5` CGTGAGCGCAACAACATCG<br>R: 5` CTCAGTTGTTCCACCCGCTT	131	60	NC_010448
FABP4	F: 5` TTCAAATTGGGCCAGGAAT<br>R: 5`ATTCTGGTAGCCGTGACACC	191	60	NM_001002817

Table S2. Expression levels of miRNAs in cell culture medium supplemented with 10% of fetal bovine serum (FBS). SE – standard error.

miRNA	Expression	SE
miR-21a	6.3	0.52
miR-92a	0	
miR-146a	0	
miR-383	0.8	0.72
miR-26b	0	
miR-30a	6.7	0.32
miR-148a	3.7	0.15
miR-199a	4.5	0.65

Table S3. Lipid droplet content and relative gene expression of *CEBPA*, *FABP4*, and *PPARG* genes across the consecutive days of the experiment (supplementary to Figure 2). The regression slope and p-values were obtained using the Locally Weighted Scatterplot Smoothing method of the ggplot2 package.

	Regression slope	Pr(>|t|)
Lipid accumulation	0.0137	0.000196 ***
CEBPA	0.03713	0.00357 **
FABP4	7.866	0.000811 ***
PPARG	1.8170	0.0114 *

Table S4. Expression levels of intracellular miRNAs over the course of differentiation. The comparisons were made between days of the experiment for one miRNA at a time, so the table should be read column-wise. The same letters indicate the absence of a significant difference (at the 0.05 level) between the expression levels. SE – standard error.

	miR-21a		miR-92a		miR-146a		miR-383	
Day	Expression	SE	Expression	SE	Expression	SE	Expression	SE
0	311037.7[a]	177776.1	130753.22[a]	229397.56	220.89[a]	113.24	93.27[ab]	17.86
2	499733.5[a]	285626.8	122560.23[a]	215023.52	393.23[a]	201.59	47.07[a]	9.01
4	978506.8[a]	559273.6	2103.51[a]	3690.46	776.39[a]	398.02	57.78[a]	11.06
6	1650918.4[a]	943595.9	2146.12[a]	3765.23	118.54[a]	60.77	70.32[ab]	13.47
8	2292898.0[a]	1310524.6	358279.79[a]	628577.32	417.17[a]	213.86	40.94[a]	9.84
10	2140759.0[a]	1223568.3	514323.56[a]	902345.41	594.95[a]	305.00	138.79[b]	26.58

	miR-26b		miR-30a		miR-148a		miR-199a	
Day	Expression	SE	Expression	SE	Expression	SE	Expression	SE
0	192918.2[a]	29326.41	53663.74[a]	9214.89	92806.22[a]	13351.41	601516.2[a]	112036.2
2	215100.0[ab]	32698.37	68626.90[a]	11784.29	113618.63[a]	16345.55	615991.2[a]	114732.2
4	223008.3[abc]	33900.55	91439.51[ab]	15701.56	220009.13[b]	31651.24	1399203.5[a]	260610.4
6	435505.0[c]	66203.18	160463.79[bc]	27554.09	391520.87[bc]	56325.49	1260419.3[a]	234761.0
8	295933.1[abc]	44986.19	115999.72[abc]	19918.93	258042.60[b]	37122.86	557527.0[a]	103842.9
10	420999.7[bc]	63998.16	239270.14[c]	41086.34	614577.00[c]	88415.08	1040886.7[a]	193871.7

Table S5. Expression levels of extracellular miRNAs over the course of differentiation. The comparisons were made between days of the experiment for one miRNA at the time, so the table should be read column-wise. The same letters indicate the absence of significant difference (at the 0.05 level) between the expression levels. SE – standard error.

	miR-21a		miR-92a		miR-146a		miR-383	
**Day**	**Expression**	**SE**	**Expression**	**SE**	**Expression**	**SE**	**Expression**	**SE**
0	2068.59[a]	1408.35	19085.39[a]	12068.23	261.59[b]	144.19	69.02[a]	17.76
2	277.89[a]	189.19	1930.39[a]	1220.64	32.21[a]	16.47	64.67[a]	16.64
4	340.91[a]	232.10	2275.61[a]	1438.93	21.37[a]	10.93	51.05[a]	13.13
6	3418.55[a]	2327.43	22065.47[a]	13952.62	27.69[a]	14.16	56.61[a]	14.56
8	1122.88[a]	764.48	9807.34[a]	6201.45	25.43[a]	13.01	54.60[a]	14.05
10	5622.02[a]	4435.01	3913.78[a]	2474.79	19.96[a]	10.21	91.31[a]	23.49

	miR-26b		miR-30a		miR-148a		miR-199a	
**Day**	**Expression**	**SE**	**Expression**	**SE**	**Expression**	**SE**	**Expression**	**SE**
0	227.02[b]	28.77	1539.17[b]	536.14	224.83[b]	38.80	1081.60[b]	174.82
2	73.28[a]	9.29	46.95[a]	16.35	77.35[a]	13.35	453.26[a]	73.26
4	200.36[b]	25.39	577.99[b]	201.33	625.33[cd]	107.90	1269.71[b]	205.23
6	480.22[c]	60.86	2038.63[b]	710.12	1201.94[d]	207.40	2747.86[c]	444.14
8	162.93[b]	20.65	965.36[b]	336.27	485.03[bc]	83.69	1091.88[b]	176.48
10	234.52[b]	29.72	436.89[b]	152.18	693.66[cd]	119.69	1249.69[b]	201.99

Table S6. Expression levels of intracellular and extracellular miRNAs. The comparisons were made between miRNAs, so the table should be read across the lines. The same letters indicate the absence of significant difference (at the 0.05 level) between the expression levels. SE – standard error.

	Cells		Medium	
	**Expression**	**SE**	**Expression**	**SE**
miR-21a	1511082[a]	772269	1253[b]	470
miR-92a	48694[a]	30015	6435[b]	3967
miR-146a	354[a]	91.71	35[b]	9.25
miR-383	69[a]	5.79	63.3[a]	6.61
miR-26b	281925[a]	20403.1	198[b]	14.4
miR-30a	106989[a]	18434	574[b]	99
miR-148a	228954[a]	19481.4	405[b]	34.4
miR-199a	927893[a]	130155	1152[b]	116

Table S7. Relationship between the expression in the medium and cells based on regression slopes for the log-transformed expression in the medium on the log-transformed expression in cells for different miRNA.

	**Estimate**	**P-value**
miR-21a	-0.40	0.17
miR-92a	-0.84	0.21
miR-146a	-0.33	0.18
miR-383	0.33	0.05
miR-26b	-0.12	0.68
miR-30a	-0.02	0.87
miR-148a	0.12	0.54
miR-199a	-0.25	0.41
